# Comparative Transcriptome Analysis Reveals Different Silk Yields of Two Silkworm Strains

**DOI:** 10.1371/journal.pone.0155329

**Published:** 2016-05-09

**Authors:** Juan Li, Sheng Qin, Huanjun Yu, Jing Zhang, Na Liu, Ye Yu, Chengxiang Hou, Muwang Li

**Affiliations:** 1 School of Biotechnology, Jiangsu University of Science and Technology, Zhenjiang Jiangsu 212018, China; 2 The Sericultural Research Institute, Chinese Academy of Agricultural Science, Zhenjiang Jiangsu 212018, China; USDA-ARS, UNITED STATES

## Abstract

Cocoon and silk yields are the most important characteristics of sericulture. However, few studies have examined the genes that modulate these features. Further studies of these genes will be useful for improving the products of sericulture. JingSong (JS) and Lan10 (L10) are two strains having significantly different cocoon and silk yields. In the current study, RNA-Seq and quantitative polymerase chain reaction (qPCR) were performed on both strains in order to determine divergence of the silk gland, which controls silk biosynthesis in silkworms. Compared with L10, JS had 1375 differentially expressed genes (DEGs; 738 up-regulated genes and 673 down-regulated genes). Nine enriched gene ontology (GO) terms were identified by GO enrichment analysis based on these DEGs. KEGG enrichment analysis results showed that the DEGs were enriched in three pathways, which were mainly associated with the processing and biosynthesis of proteins. The representative genes in the enrichment pathways and ten significant DEGs were further verified by qPCR, the results of which were consistent with the RNA-Seq data. Our study has revealed differences in silk glands between the two silkworm strains and provides a perspective for understanding the molecular mechanisms determining silk yield.

## Introduction

*Bombyx mori* was domesticated from the wild silkworm (*B*. *mandarina*) and has been used in various economic applications for thousands of years. Indeed, silk produced by domestic silkworms is widely used as a textile material in daily life [1,2]. However, little is known about the molecular mechanisms contributing to different silk yields in domestic silkworm strains [3].

Hundreds of silkworm strains are preserved at the Sericultural Research Institute, Chinese Academy of Agricultural Sciences, and hereditary and ecological divergence of various genes has been shown to be responsible for the quality of silk produced by silkworm in many strains. Jingsong (JS) and Lan10 (L10) are typical strains with high and low silk yields, respectively. The JS strain is widely used in various applications as a commercial strain and has silk properties that are advantageous for silk yields. In contrast, L10 produces smaller cocoons and has poor silk-producing properties [4]. The silk yield of JS is about five-fold higher than that of L10. The silk yield is about 0.5 g per individual in Js, while that of L10 is about 0.1 g. Because cocoon and silk yields are determined by genetic quantitative traits, molecular marker techniques have been used widely in silkworms in order to identify many quantitative trait loci (QTLs) [5–7]; indeed, our laboratory has used such techniques to identify 14 QTLs [4]. Although technopositioning can be useful for finding the genetic loci of large fragments having relatively high location-dependent (LOD) values, RNA-Seq based transcriptome analysis may be more suitable for detecting minor genes [8,9].

The silk gland (SG), which is the primary organ responsible for silk yield, can be divided into three compartments: the anterior silk gland (ASG), middle silk gland (MSG), and posterior silk gland (PSG). The silk fibers are composed of two types of major silk proteins, fibroins and sericins, which are produced by the SGs. Fibroins and sericins are synthesized by the PSG and MSG, respectively [10]. Fibroins are composed of the fibroin heavy chain (fib-H), fibroin light chain (fib-L), and p25 proteins. The three proteins form a hexameric complex in a 6:6:1 ratio [11]. The mRNA levels of these genes and the corresponding fibroin protein accumulate in SGs during different developmental stages [12]. The lumen of the MSG contains more than seven major sericins and various uncharacterized minor proteins, which are encoded mainly by *Ser1*, *Ser2*, *Ser3*, *MSGS*^*3*^, *MSGS*^*4*^, and *MSG*^*5*^ [13–18]. However, no studies have examined the molecular mechanisms in SGs that contribute to different silk yields in domestic silkworms.

In this study, we used the SGs from third day fifth-instar larvae of the JS and L10 strains to examine the silk yield differences between the two strains by RNA-Seq. Our results will provide insights into the molecular mechanisms of silk production and lead to improved cocoon silk yield [19,20].

## Methods

### Sample preparation

L10 and JS strains, which have different rates of silk production, were chosen from the silkworm resource library. The larvae were reared on live mulberry under a constant 14-h light/10-h dark photoperiod at 25 ± 1°C and 75% ± 3% relative humidity. Intact SGs were dissected and frozen in liquid nitrogen immediately for RNA-seq.

### RNA extraction and library preparation

SG total RNAs were extracted using TRIzol reagent (Invitrogen, USA) following the manufacturer's protocol. An Agilent 2100 Bioanalyzer was used to determine RNA quality. Contaminating genomic DNA was removed from a 3 μg total RNA aliquot by treatment with 10 μg DNase I (Takara, Japan) for 30 min at 37°C. RNA was purified from the digest using Dynabeads^®^ Oligo (dT) 25 (Life Tech, USA). Finally, 100 ng purified mRNA per sample were used to construct the respective cDNA libraries using an NEBNext^®^ UltraTM RNA Library Prep Kit for Illumina (NEB, USA).

### RNA sequencing (RNA-Seq) and quality control

The clusters for RNA-Seq were generated using 10 ng cDNA of each sample–with a TruSeq PE Cluster Kit (Illumina, USA). RNA-Seq consisting of 100 bp paired-end reads was performed on an Illumina HiSeq 2500 platform (Illumina, San Diego, CA, USA) with read lengths The quality of the raw data was assessed using the FastQC and FastX-Toolkit [21,22]. Adaptor sequences, unknown sequences (N), and low-quality reads and their paired sequences <50 bp were removed from the raw data. All of the downstream analyses were based on high-quality, clean sequences. The clean reads were directly mapped to the silkworm reference genome [23] using the Bowtie short read aligner [24].

### Analysis of differentially expressed genes (DEGs)

The expression levels of genes were normalized using the reads per kb per million reads (RPKM) method [25]. The gene expression levels were compared between the two strains using DEGSeq [26]. *P* values were adjusted for multiple comparisons using the Benjamini and Hochberg method [27]. DEGs were filtered with the following thresholds: false-discovery rate (FDR) ≤ 0.001 and |log2 Fold Change (Log_2_ FC)| ≥ 1.

### Annotation and enrichment analysis for gene ontology (GO) and KEGG analyses

All genes in the current transcriptome of the silkworm were annotated based on BLAST homology searches. The expressed genes were searched against the Swiss-Prot and TrEMBL databases by double-direction BLAST with an e-value of 1e^-5^ or less. Orthologous genes were then annotated to GO terms and KEGG pathways using GOPipe [28] and the KEGG database [29]. To explore the function of DEGs between the two silkworm strains, GO and KEGG enrichment analyses were performed using hypergeometric tests. An FDR of 0.05 or less was set as the threshold for screening the significantly enriched GO and KEGG terms.

### Validation by quantitative real-time reverse transcription polymerase chain reaction (qRT-PCR)

To further validate the RNA-Seq results, SGs from three biological samples for each strain were dissected from 3-day-old fifth instar larvae and divided into three parts (ASG, MSG, and PSG). In addition to the SGs, the fat body, midgut and hemolymph were also dissected. RNAiso Plus (TaKaRa Dalian, China) was used to isolate total RNA according to the manufacturer’s instructions. Gel electrophoresis and ultraviolet spectrophotometry were used to determine the integrity and purity of the RNA. One microgram of total RNA from each sample was used to synthesize cDNA using a PrimeScript^™^ RT reagent Kit with gDNA Eraser (Perfect Real Time, TaKaRa)—followed by storage at -20°C. Real-time quantitative PCR was carried out in a reaction volume of 20 μL, containing 2 μL of template, 10 μL of 2× SYBR Premix EX Taq (TaKaRa), 0.4 μL of 50× ROX Reference Dye (TaKaRa), and 0.4 μL of specific primers (10 μM). The PCR amplification efficiency (E) and R^2 of each primer pair was calculated from the slope of a standard curve, which was conducted according to MIQE (Minimum information for publication of quantitative real-time PCR experiments) guidelines [30]. The qRT-PCR primer sequences, which were designed based on the consensus sequence of each alignment, and their efficiencies are provided in S1 Table. qRT-PCR was performed with an ABI7300 real-time PCR system, using the following conditions: 95°C for 5 min, followed by 40 cycles of 95°C for 5 s, 60°C for 31 s and dissociation.

The mRNA quantity of each gene was calculated with the 2^-△△Ct^ method [31] and normalized to the abundance of the house-keeping gene *BmGAPDH* (Accession No. XM_012690444) [32]. The relative mRNA levels of each gene are presented as fold changes relative to the expression level of *BmGAPDH*.

The expression levels of each gene in the two respective strains were compared based on a Student's T-test. Differences in gene expression between the two strains were considered significantly different at *P* ≤ 0.05.

## Results

### Overview of transcriptome sequencing data

Pooled total RNAs were isolated from 3-day-old fifth instar larval SGs corresponding to either the JS or L10 strain of silkworm. cDNA libraries were then constructed and sequenced using an Illumina HiSeq2500 platform. 22,704,312 and 21,167,022 raw reads were generated from the cDNA libraries. The quality of the RNA-Seq data is listed in the S2-1 Table. In total, 22,635,272 and 21,099,316 clean reads were filtered from the raw data. The clean data has been submitted to the NCBI SRA database with the accession numbers: SRR3190017 and SRR3190036. These clean reads were mapped to reference genome sequences obtained from the Silkworm Genome Database (Silk DB; https://www.silkdb.org/silkdb/) using the Bowtie short read aligner [23,24]. A total of 14,772,549 (65.26%) clean reads were mapped to 10,849 genes in JS, and 13,787,558 (65.35%) clean reads were mapped to 11,218 genes in L10 (S2-2 Table). There were 10,097 commonly expressed genes between the two strains with ~ 47% and 56% of the genes covered by more than 90% of the clean reads in the JS and L10 strains, respectively (S1 Fig).

### DEGs in SGs

The gene expression levels were compared between the two strains using DEGSeq [26]. Based on the thresholds for screening DEGs, 1411 genes were identified as DEGs in JS compared to L10; of these, 738 were up-regulated, and 673 were down-regulated in JS. All of the DEGs are listed in S3 Table.

### KEGG and GO annotations and enrichment analyses

**Fig 1:**
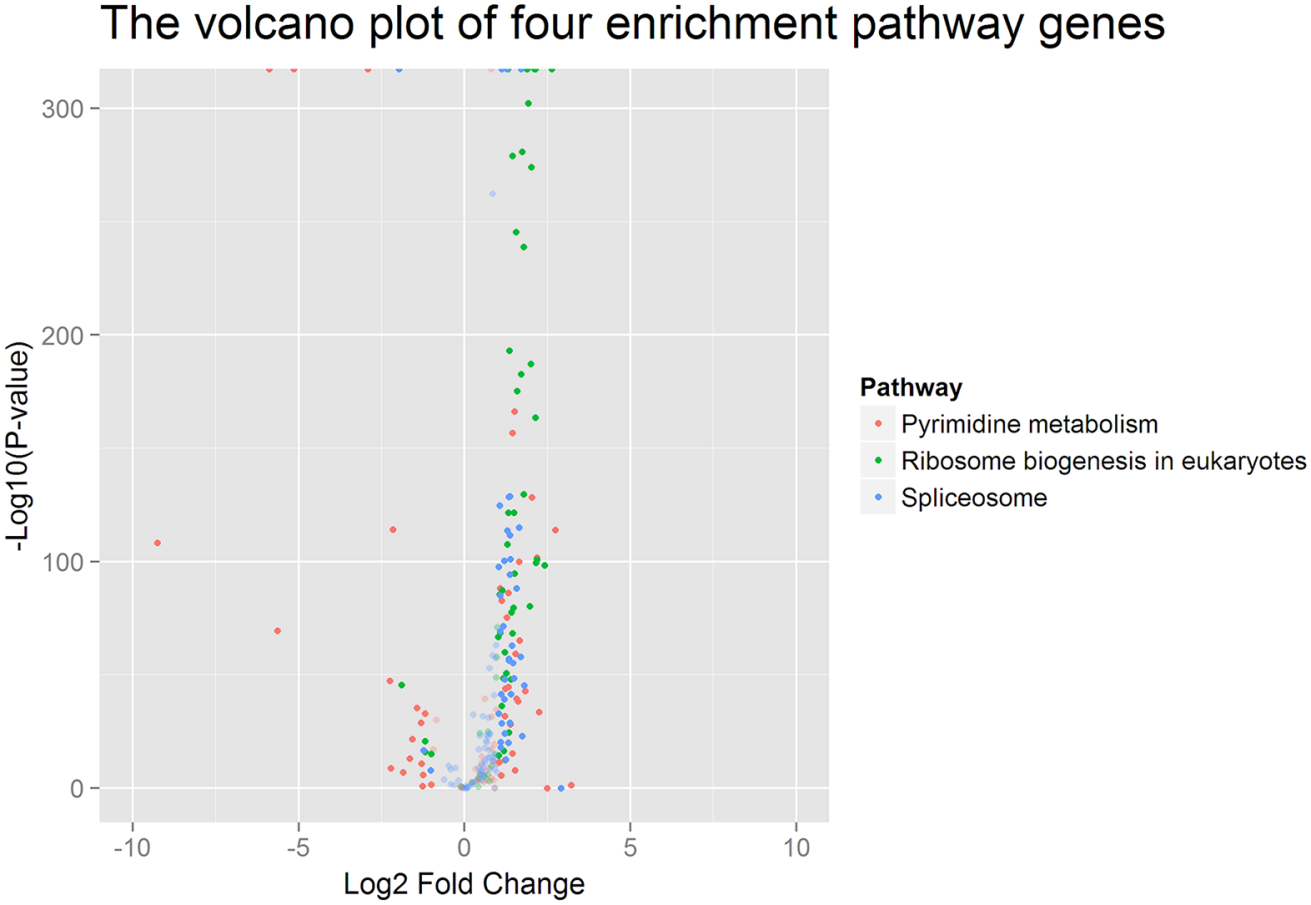
Volcano plot of DEGs in enrichment pathways. The pink points indicate genes in the pyrimidine metabolism pathway, green points indicate the genes in ribosome biogenesis in eukaryotes, and blue points indicate genes in the spliceosome pathway. The translucent points indicate genes for which the log2 fold change (Log2 FC) was less than 1 (and are therefore not differentially expressed).

Based on homologous proteins identified in BLAST searches, 2551 genes were mapped to 32 pathways comprising the five main KEGG database categories (S2 Fig). KEGG pathway enrichment analysis was performed based on hypergeometric tests. The DEGs between the two silkworm stains were significantly enriched in three pathways (Table 1), pyrimidine metabolism, ribosome biogenesis in eukaryotes and the spliceosome. These genes are mainly responsible for protein production. Compared with L10, more than 97% of DEGs in JS were significantly up-regulated in ribosome biogenesis in eukaryotes and the spliceosome (Fig 1). Additionally, ~72% of DEGs in JS were also significantly up-regulated compared with those in L10 in the pyrimidine metabolism pathway. These results suggested that the processing and biosynthesis of proteins were activated to a greater degree in JS than in L10. All DEGs in the enrichment pathways are listed in S4 Table.

**Table 1:** KEGG enrichment of the DEGs between L10 and JS.

KEGG pathway	Cluster frequency	Genome frequency of use	*P* value	FDR
Ribosome biogenesis	39/ 426 genes	60 / 2551 genes	3.40E-10	6.94E-08
Spliceosome	35/ 426 genes	100 /2551 genes	1.96E-04	3.96E-02
Pyrimidine metabolism	29 / 426 genes	75/ 2551 genes	1.82 E-04	3.69E-02

Cluster frequency is the number of DEG annotation terms relative to the ontology; genome frequency of use is the number of annotations to the reference genes of the term relative to the ontology.

It is reasonable to hypothesize that silk fiber biosynthesis is dependent on the production of proteins in the SG. The results of GO enrichment analysis reflected the differences in the SG between the two strains. The GO terms for the DEGs were annotated using GOPipe [28], and the GO classification bar graph was drawn according to the GO IDs. From this analysis, 4110 genes were annotated with 51 GO terms across three main categories (S3 Fig). The DEGs between the two silkworm stains were significantly enriched in nine GO items (Table 2). The dominant GO term was membrane-enclosed lumen, belonging to the cellular component, which is consistent with results from the pathway enrichment analysis. One of the DEG enriched pathways was ribosome biogenesis. Ribosomes are mainly attached to the membrane-enclosed lumen, for example the endoplasmic reticulum. It indicated that the protein process had significant differences between the L10 and JS SGs. The DEGs were also enriched in some biological processes, including metabolic processes, cellular component biogenesis, cell wall organization or biogenesis, and cellular process. We observed significant differences in DEGs involved in molecular functions for nutrient reservoir activity and catalytic activity between the two strains. The results implied that most of the DEGs may play a positive role in protein biosynthesis.

**Table 2:** Gene ontology enrichment of the DEGs between L10 and JS.

GO item	Cluster frequency	Genome frequency of use	*P* value	FDR
membrane-enclosed lumen	117 /585 genes	421 /4110 genes	3.54E-15	1.81E-13
organelle	367 / 585 genes	2367 /4110 genes	2.75E-3	2.09E-02
organelle part	223 / 585 genes	1194 /4110 genes	1.25E-07	3.20E-06
metabolic process	359 / 585 genes	2242 / 4110 genes	1.38E-04	2.34E-03
cellular component biogenesis	63/ 585 genes	300 /4110 genes	3.35E-04	4.27E-03
cellular process	424 / 585 genes	2809 / 4110 genes	8.42E-03	4.77E-02
cell wall organizationor biogenesis	2 / 585 genes	3 / 4110 genes	2.87E-03	2.09E-02
nutrient reservoir activity	3 / 585 genes	6 / 4110 genes	4.80E-03	3.06E-02
catalytic activity	273 / 585 genes	1695/4110 genes	1.80E-03	1.83E-02

Cluster frequency is the number of DEG annotation terms relative to the ontology; genome frequency of use is the number of annotations to the reference genes of the term relative to the ontology.

### Validation of DEGs by qRT-PCR

**Fig 2:**
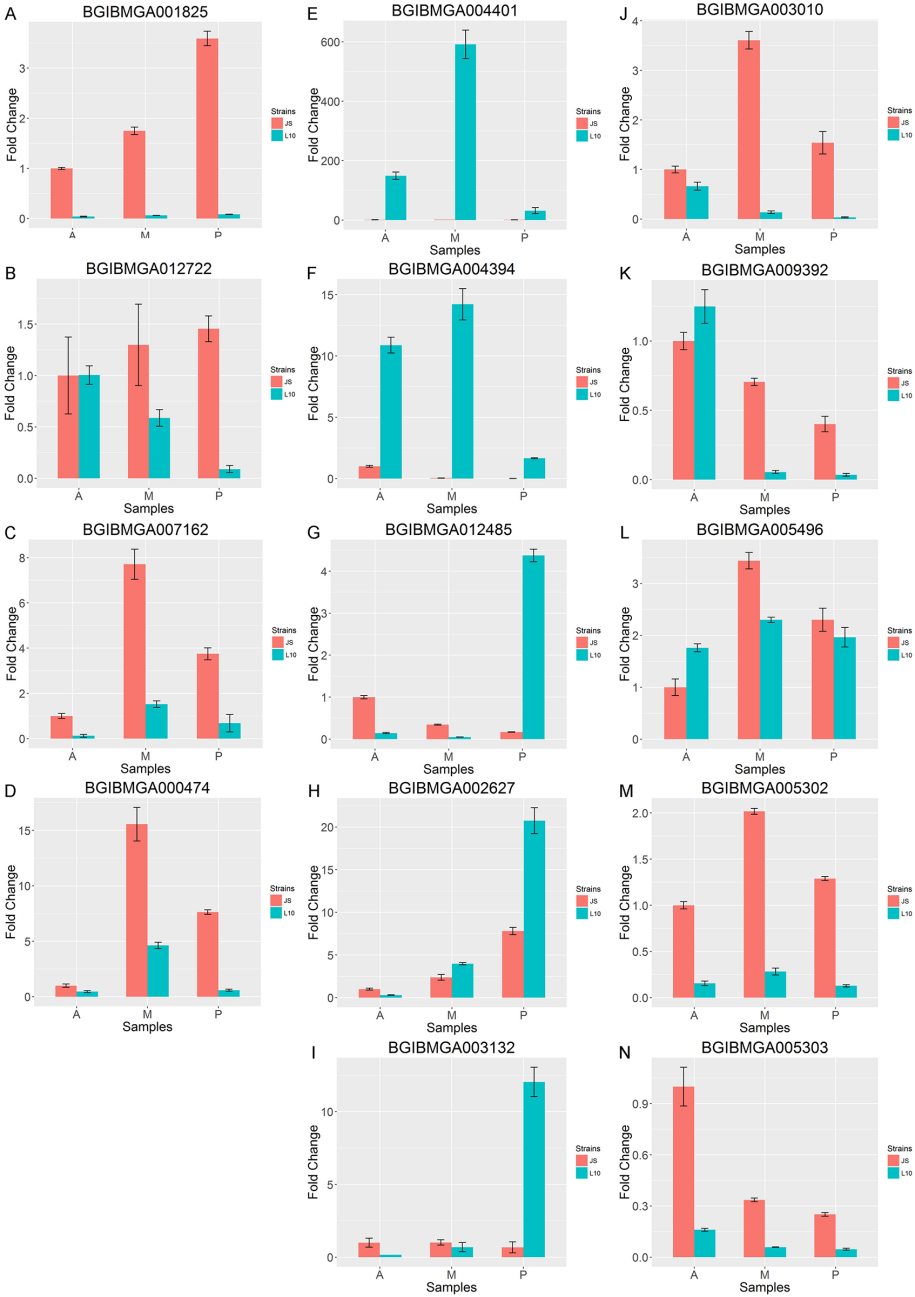
Quantitative real-time PCR validation for genes. Red bars indicate gene expression in JS and blue bars indicate gene expression in L10. A and B are related to spliceosome enriched transcripts, C and D are involved in ribosome biogenesis. Transcripts in JS were significantly higher than L10 between each comparison pair. Genes of the second column (E-I) were with higher expression of SGs in L10. Genes of the third column (J-N) were with higher expression of SGs in JS. **A:** the anterior SG in L10 or JS; **M:** the middle SG in L10 or JS; **P:** the posterior SG in L10 or JS.

To validate the RNA-Seq data, qPCR was performed for representative genes in the enrichment pathways and 10 additional significantly different genes selected from the DEGs. The gene sequences were obtained from the silkworm genome sequence [33]. The qPCR expression results were similar to the results obtained from the Illumina sequencing data. In enrichment pathways, the expressions of three representative genes in JS were significantly higher than that in L10 (*P* ≤ 0.05, Fig 2, Table 3). The expression of *BGIBMGA012722* in JS was significantly higher than that in the L10 PSG. For the other 10 DEGs, genes in the second column of Fig 2 were up-regulated in the L10 SG compared to JS (*P* ≤ 0.05, Fig 2E~2I, Table 3). In contrast, the genes in the third column of Fig 2 were more highly expressed in JS SG (*P* ≤ 0.05, Fig 2J~2N, Table 3).

**Table 3:** Comparison of NSG and qPCR results in SG between L10 and JS.

Genes	qPCR results						RNA-Seq results	
	Log2 FC of ASG	*P* of ASG	Log2 FC of MSG	*P* of MSG	Log2 FC of PSG	*P* of PSG	Log2 FC	*P*
BGIBMGA004401	-7.1399	2.30E-03*	-7.9782	2.20E-03*	-5.1589	3.35E-02*	-9.0479	4.06E-97**
BGIBMGA004394	-3.4437	1.30E-03**	-8.4648	2.70E-03**	-8.5836	3.00E-04**	-9.1367	0.00E+00**
BGIBMGA012485	2.78715	1.00E-04**	2.7971	3.00E-04**	-4.6970	4.00E-04**	-2.4091	0.00E+00**
BGIBMGA002627	1.6957	6.30E-03**	-0.7342	8.10E-03**	-1.4118	2.70E-03**	-8.4626	1.11E-273**
BGIBMGA003132	1.9319	9.33E-02	0.4982	3.85E-01	-4.0509	7.00E-04**	-9.6503	1.25E-68**
BGIBMGA003010	0.5889	5.6E-03**	4.7015	7.00E-04**	5.4603	7.30E-03**	3.6471	3.34E-68**
BGIBMGA009392	-0.3239	5.01E-02	3.6700	0.00E+00**	3.4948	6.40E-03**	5.1310	9.21E-95**
BGIBMGA005496	-0.8031	6.10E-03**	0.5807	3.40E-03**	0.2268	1.19E-01	3.9439	8.24E-164**
BGIBMGA005302	2.6819	0.00E+00**	2.8278	0.00E+00**	3.3275	0.00E+00**	3.1219	0.00E+00**
BGIBMGA005303	2.6394	5.80E-03**	2.5058	4.00E-04**	2.5058	0.00E+00**	3.3138	0.00E+00**
BGIBMGA001825	4.6678	0.00E+00**	4.8072	6.00E-04**	5.4342	5.00E-04**	1.6915	1.03E-58**
BGIBMGA012772	0.0460	8.95E-01	1.1738	7.70E-02	3.9566	1.50E-03**	1.1157	0.00E+00**
BGIBMGA007162	2.8818	1.00E-03**	2.3362	2.70E-03**	2.2881	0.00E+00**	2.1343	3.47E-164**
BGIBMGA000474	1.0836	1.13E-02*	1.7528	5.00E-03**	3.6910	0.00E+00**	2.1416	0.00E+00**

Log2 FC is referred to log2 fold change and *P* is referred to *P* value. *P* ≤ 0.05 is marked with "*" and *P* ≤ 0.01 is marked with "**".

Based on the qRT-PCR results, we found that the following genes were differentially expressed in the SGs, fat, midgut, and hemolymph (S4 Fig). Compared with JS, genes with higher expression in the SG of L10, most of which were more highly expressed in the fat body (*BGIBMGA004401* and *BGIBMGA004394*) and some of which were expressed at significantly higher levels in the midgut (*BGIBMGA002627* and *BGIBMGA003132*). Compared with L10, genes with higher expression in the SG of JS silkworms, were also expressed highly in the fat body, however, expression of the genes was lower than that observed in the hemolymph of L10 silkworms. As described above, the expression patterns of DEGs in SGs was different from that of fat body and hemolymph in L10 and JS. Moreover, we predicted that the biosynthesis and secretion of silk proteins may depend on the cooperation of multiple tissues. The expression profiles of these genes across six tissues are shown in S4 Fig.

## Discussion

### Enrichment pathways in SGs were mainly related to protein synthesis

Ribosome biogenesis is necessary for cellular adaptation, growth, and proliferation and provides a major source of energy and biosynthesis for cells [34]. DEGs enriched in the ribosome biogenesis pathway suggested a relationship between protein expression and efficiency in silk protein synthesis. Ribosome biogenesis underlies the cell’s capacity to grow because cell growth, or increases in cell mass, requires large numbers of ribosomes, the molecular factories that carry out protein synthesis [35]. The high expression of ribosome proteins reflects the rapid biosynthesis in SGs, and the SG size of Js is much bigger than that of L10. These phenomena might correspond with the expression of DEGs in JS and L10. Moreover, 39 genes were enriched in the pathway of ribosome biogenesis, most of which were up-regulated in JS. For example, the expression level of *BGIBMGA007162* and *BGIBMGA000474* in JS were exhibited up to four times fold change in MSG and PSG than the expression level of those genes in L10 (Fig 2C~2D), and these up-regulated genes that may result in more formation of silk fiber in JS than L10. On the other hand, ribosomal proteins are not only essential in the assembly of translational machinery but are also crucial for the transport of primary proteins [36]. Additionally, the spliceosome is also associated with protein synthesis through catalysis of pre-mRNA splicing [37,38]. Zhou et al predicted that Bm *eIF5A*, which is involved in cell proliferation, had two spliceosome sites and may be associated with silk expression in *Bombyx mori* [39]. Indeed, our results showed that DEGs were enriched in the spliceosome pathway in the SG, suggesting that genes related to silk yield may facilitate the expression of silk protein.

### DEGs up-regulated in L10

We characterized 10 DEGs, five of which were up-regulated in L10, and five of which were up-regulated in JS (Fig 2 and S4 Fig). We manually annotated the functions of these 10 DEGs. In L10, genes encoding a 30 kDa lipoprotein and phosphoserine aminotransferase were up-regulated. The gene encoding the 30 kDa lipoprotein is expressed mainly in the fat body and integument during the larval and pupal stages [40,41] and is involved in various physiological processes, including energy storage, embryonic development, and immune response in silkworms [42–45]. The 30 kDa protein is involved in the degeneration of the *B*. *mori* SG by a caspase-dependent pathway [46]. Thus, the highly efficient biosynthesis and secretion of silk proteins may be suppressed by the 30 kDa lipoprotein. We also identified the *BGIBMGA012485* gene, which functions as a phosphoserine aminotransferase and is involved in serine biosynthesis [47,48], as being up-regulated in the PSG but not the ASG or MSG of L10; the opposite expression pattern was observed in JS. We speculated that this gene may be regulated by another component that could specifically block production in the MSG and ASG in L10. As we known, the spinneret is another important tissue for spinning. Filippi's glands (FG), which are a pair of tiny glands located at the distal ends of the two ASGs, join to form a common duct. Spinneret and FGs contribute to silk formation, spinneret-expressed genes are closely related to silk formation and provide a suitable environment for silk fiber formation. In a previous study, FGs were suggested to possibly function in transporting small solutes to the ASG duct [49]. DEGs in the spinneret and FG between day 3 of fifth instar larvae and wandering stage larvae were mainly enriched in the pathways associated with ion-transport, chitin binding, and energy metabolism. But these findings are different from our study in which it is mainly related to pyrimidine metabolism, ribosome biogenesis in eukaryotes, and the spliceosome. That might be because the SG is responsible for biosynthesis of the silk ingredients and the spinneret and FG provide the environment for mixing the ingredients. It suggests that spinneret, FG and SG play different functional roles during the silk fiber formation. Despite high expression in the PSG of L10, the silk yield was low. Thus, of the up-regulated DEGs in the L10, some may play important roles in defense against pathogens and in low efficiency of protein synthesis. Additionally, some of the genes may be involved in signaling processes during cell communication.

### DEGs up-regulated in JS

We identified a number of genes that were up-regulated in JS, including a gene (*BGIBMGA003010*) encoding a protein with similar function to Werner helicase-interacting protein 1 (WRNIP1). *BGIBMGA003010* encodes a protein that exhibits ATPase activity and is involved in DNA synthesis and WRNIP1 has been reported to function in the metabolism of ubiquitinated proteins [50]. Several different DNA repair pathways have been shown to be regulated by ubiquitination. For example, the proper execution of nucleotide excision repair depends upon the polyubiquitination and degradation of a subset of its component enzymes [51,52], whereas translesion DNA synthesis is regulated by mono- and polyubiquitination of *PCNA3* [53]. The *BGIBMGA009392* gene encodes a protein with protein kinase functionality, which is fundamental to most signaling and regulatory processes in eukaryotic cells [54]. The DESs up-regulated in JS are mainly enriched in the pyrimidine metabolism, spliceosome and ribosome biogenesis pathways. The up-regulatory genes in the pyrimidine metabolism and spliceosome pathways will facilitate the transcription process of mRNAs which are required during the silk fiber synthesis and the highly expressed genes in ribosome biogenesis pathway will benefit the translation process of silk related proteins. The more proteins are translated, the more silk fiber will be produced. That might be the cause which led to the difference in silk production between two silkworm strains.

## Supporting Information

S1 Fig The proportions of genes covered by clean reads in L10 and JS (TIF)

S2 Fig Annotated KEGG pathways in SG transcriptome data. (TIF)

S3 Fig Annotated GO terms in SG transcriptome data. (TIF)

S4 Fig Quantitative real-time PCR validation for genes in six tissues. **A:** the anterior SG in L10 or JS; **F:** the fat of L10 or JS; **M:** the middle SG in L10 or JS; **P:** the posterior SG in L10 or JS; **X:** the haemolymph of L10 or JS; **ZC:** the midgut of L10 or JS.
(TIF)

S1 Table Primer sequences used for the qPCR validation experiment. (XLSX)

S2 Table Summary of Illumina sequencing and reads mapping. (XLSX)

S3 Table DEGs between L10 and JS. (XLSX)

S4 Table The DEGs in enrichment pathways. (XLSX)
