# Supplementary figures and images for: Comparative Transcriptome Analysis Reveals Different Silk Yields of Two Silkworm Strains
Source: PLoS One. 2016 May 9;11(5):e0155329. doi: 10.1371/journal.pone.0155329 (PMC4861282; doi:10.1371/journal.pone.0155329)

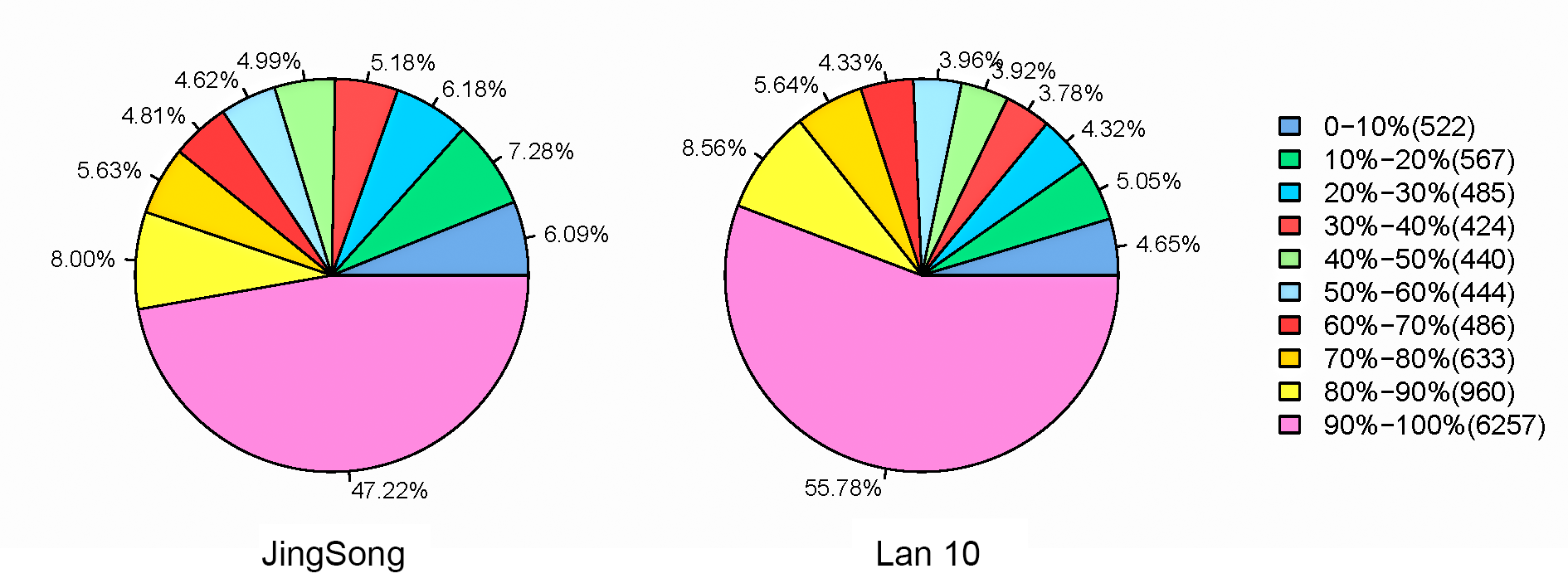

Supplement: S1 Fig — (TIF) [file pone.0155329.s001.tif]

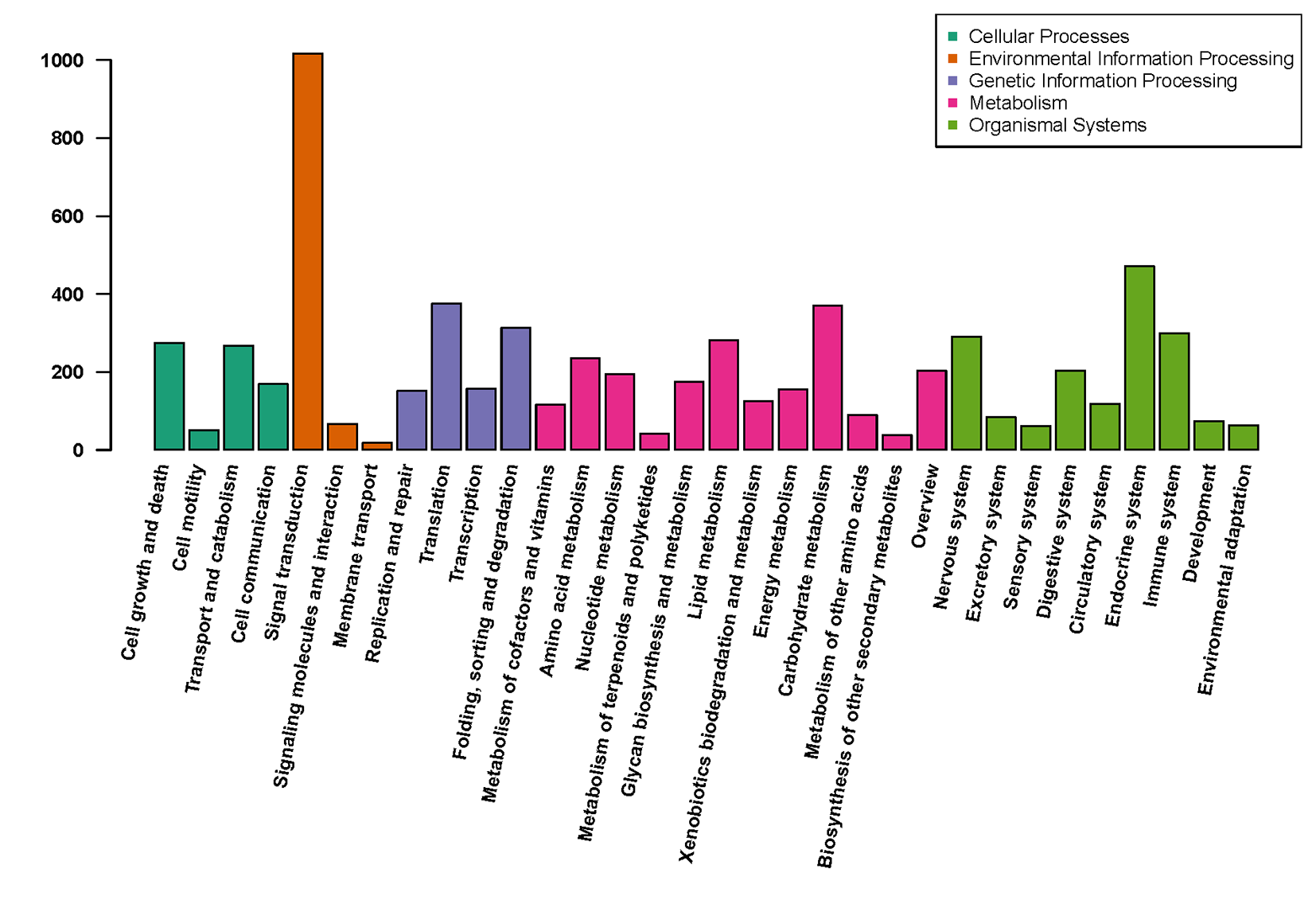

Supplement: S2 Fig — (TIF) [file pone.0155329.s002.tif]

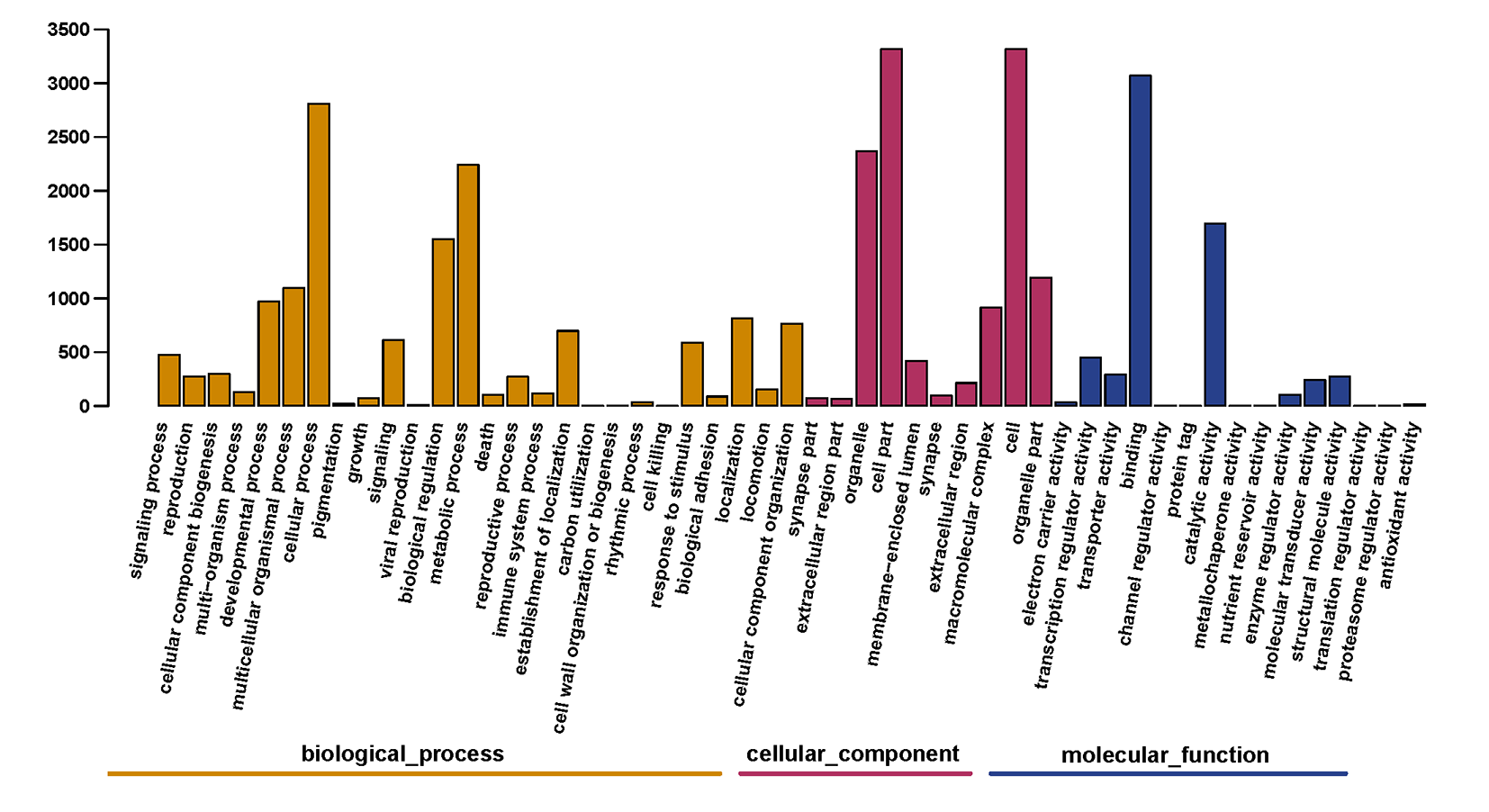

Supplement: S3 Fig — (TIF) [file pone.0155329.s003.tif]

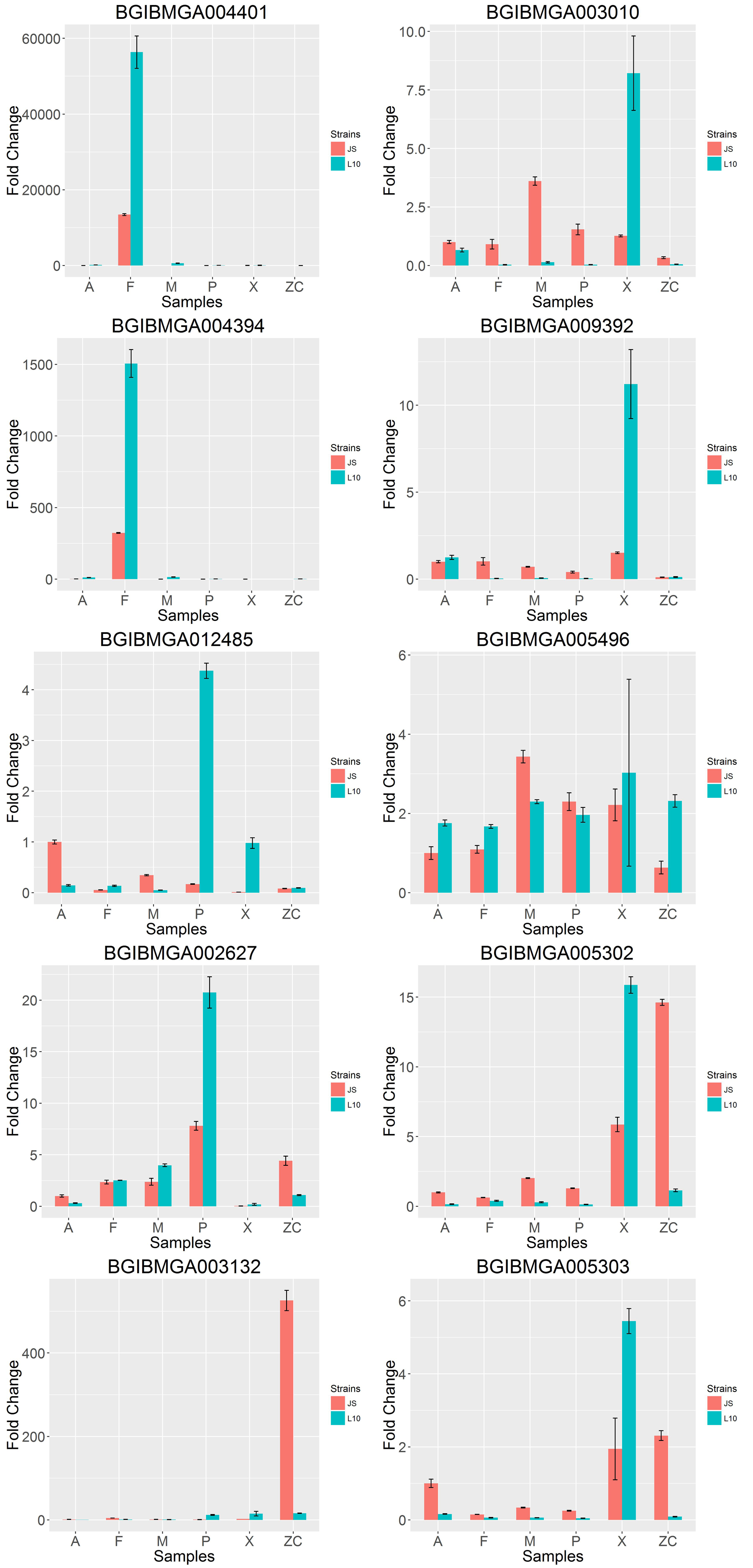

Supplement: S4 Fig — A: the anterior SG in L10 or JS; F: the fat of L10 or JS; M: the middle SG in L10 or JS; P: the posterior SG in L10 or JS; X: the haemolymph of L10 or JS; ZC: the midgut of L10 or JS. (TIF) [file pone.0155329.s004.tif]
